# Use of Whole-Genome Sequencing to Explore *Mycobacterium tuberculosis* Complex Circulating in a Hotspot Department in France

**DOI:** 10.3390/microorganisms10081586

**Published:** 2022-08-06

**Authors:** Typhaine Billard-Pomares, Julie Marin, Pauline Quagliaro, Frédéric Méchaï, Violaine Walewski, Samira Dziri, Etienne Carbonnelle

**Affiliations:** 1 Groupe Hospitalier Paris Seine Saint-Denis, AP-HP, Hôpital Avicenne, Service de Microbiologie Clinique, 93000 Bobigny, France; 2 INSERM UMR 1137, IAME, Université Sorbonne Paris Nord, 75018 Paris, France; 3 Groupe Hospitalier Paris Seine Saint-Denis, AP-HP, Hôpital Jean Verdier, Service de Pédiatrie, 98140 Bondy, France; 4 Groupe Hospitalier Paris Seine Saint-Denis, AP-HP, Hôpital Avicenne, Service de Maladies Infectieuses et Tropicales, 93000 Bobigny, France

**Keywords:** *Mycobacterium tuberculosis*, whole-genome sequencing, hotspot region, lineage diversity

## Abstract

The Seine-Saint-Denis is the French metropolitan department with the highest incidence of tuberculosis (TB). Our aim was to explore epidemiological and phylogenetic characteristics of TB strains in this hotspot department. We performed WGS on 227 strains of *Mycobacterium tuberculosis complex* isolated from patients at the Avicenne Hospital from 2016 to 2021 and randomly selected to represent the clinical diversity of French TB localization. Clinical and demographic data were recorded for each TB patient. The mean age of patients was 36 years old. They came from Africa (44%), Asia (27%), Europe (26%) and America (3%). Strains isolated from extrapulmonary samples were associated with Asian patients, whereas strains isolated from pulmonary samples were associated with European patients. We observed a high level of lineage diversity in line with the known worldwide diversity. Interestingly, lineage 3 was associated with lymph node TB. Additionally, the sensitivity of WGS for predicting resistance was 100% for rifampicin, isoniazid and ethambutol and 66.7% for pyrazinamide. The global concordance with drug-susceptibility testing using the phenotypic approach was 97%. In microbiology laboratories, WGS turns out to be an essential tool for better understanding local TB epidemiology, with direct access to circulating lineage identification and to drug susceptibilities to first- and second-line anti-TB drugs.

## 1. Introduction

Worldwide, tuberculosis (TB) remains one of the most prevalent infectious diseases and has become the leading infectious disease killer in the world. In 2019, the World Health Organization (WHO) estimated 10 million new cases of active TB [1]. TB is caused by etiological agents belonging to the *Mycobacterium tuberculosis* complex (MTBC). This complex includes different human and animal TB species.

The complete genome of the *M. tuberculosis* reference strain H37Rv was described in 1998 [2]. The total genome was 4.41 million base pairs in length and composed of approximately 4000 genes. Since then, thanks to advances in DNA sequencing technologies, whole-genome MTBC sequences have been generated from around the world [2]. Whole-genome sequencing (WGS) is now an essential tool in epidemiologic and evolutionary studies of TB. Compared to the phenotypic approach, which is widely used in hospital laboratories, WGS can rapidly detect a large panel of drug resistance genes simultaneously. This genomic approach could provide more information than the phenotypic approach for clinical management of TB patients, especially multidrug-resistant tuberculosis MDR-TB patients, predicting resistance to first- and second-line anti-TB drugs [3,4]. Moreover, WGS can obtain interesting information on the genomic characteristics of the strains in terms of virulence and phylogenetic lineage [5].

The global population structure of *M. tuberculosis* isolates is classified into seven major lineages with a strong phylogeographic structure [6,7], including the Indo-Oceanic lineage (lineage 1), the East Asian lineage (lineage 2), the East-African-Indian lineage (lineage 3), the Euro-American lineage (lineage 4), the West African-1 lineage (lineage 5) and the West African-2 lineage (lineage 6), distinct from the *M. bovis* clade [4]. More recently, a new phylogenetic lineage of MTBC, referred to as lineage 7, has been found in northwest Ethiopia [8]. Identifying *M. tuberculosis* lineage is essential to better control of TB due to the effect of the genotype diversity on disease outcome [9]. For example, several clinical studies have reported that lineage 2 was associated with higher severity and increased treatment failure even in the absence of drug resistance [10,11]. A study showed that lineage 3 was able to evade the immune response of the host, which contributes to the persistence and the potential occurrence of outbreaks due to this lineage in the human population [12].

Notified cases of TB have decreased steadily for decades. France represents a low-incidence country, with a rate of 7 notified TB cases per 100,000 inhabitants, [13] but there are important territorial disparities. For instance, in the department of Seine-Saint-Denis, the incidence of TB is 27/100,000 inhabitants. This territory is the French metropolitan department with the highest incidence of TB, also characterized by a high level of social deprivation indicators [14]. However, to our knowledge, the local diversity of strains circulating in the department of Seine-Saint-Denis has never been investigated. The high incidence of TB in this department constitutes a major public health concern and prompted us to explore the epidemiological and phylogenetic characteristics of the strains isolated in the hospital laboratory of Avicenne, located in this department. 

In this study, we whole-genome-sequenced 227 MTBC strains (i) to investigate the local epidemiology and phylogenetic characteristics of TB strains and (ii) to evaluate the performance of the WGS technology for determining drug susceptibility and resistance compared to standard phenotypic drug-susceptibility testing (DST) for isoniazid (INH), rifampicin (RIF), pyrazinamide (PZA) and ethambutol (EMB). 

## 2. Materials and Methods

### 2.1. Ethics Statement and Study Population

We conducted a retrospective study that was approved by the Local Ethics Committee for Clinical Research of the Paris Seine-Saint-Denis University Hospitals under no. CLEA-2021-185. Clinical and demographic data were recorded for each TB patient. The sociodemographic data collected included sex, age and country of origin of the patients. Countries of birth were assigned to five world regions according to Gagneux et al. [6], with the different sub-regions of Africa and Asia pooled into two individual groups, Africa and Asia.

### 2.2. Culture and Identification of MTBC

In total, 227 clinical MTBC strains taken from 222 patients during routine care at Avicenne Hospital between 2016 and 2021 were randomly selected (representing 29% of the total strains isolated at the laboratory). Strains were selected to represent the clinical diversity of French TB localization [15]. The clinical diversity of TB in France is represented as 68% for pulmonary forms and 32% for exclusively extrapulmonary forms. Thus, 136 strains isolated from pulmonary specimens and 91 isolated from extrapulmonary specimens were included to match the known distribution. Strains were isolated using Coletsos media (BioRad^®^, Marnes-la-Coquette, France). Identification of MTBC was performed using the Bioline TB Ag MPT64 Rapid test (Abbott, Chicago, IL, USA). After extracting the DNA, we confirmed TB using Hain GenoType^®^MTBDR*plus* technology (Biocentrics^®^, Bandol, France).

### 2.3. Phenotypic Drug-Susceptibility Testing

Phenotypic DST for the four first-line drugs was performed using the liquid culture system BACTEC™ Mycobacterial Growth Indicator Tube™ (MGIT 960) (Becton Dickinson^®^, Sparks, MD, USA) according to the manufacturer’s instructions, at the following critical concentrations: RIF, 1.0 mg/L; INH, 0.1 mg/L; PZA, 100.0 mg/L; EMB, 5.0 mg/L. According to the laboratory’s routine procedure, all phenotypic results showing a resistance were checked in a second experiment. The gold standard used in our study was the phenotypic DST. Sensitivity (prediction of antibiotic resistance), specificity (prediction of antibiotic susceptibility), negative predictive value and positive predictive value of WGS were determined for each antibiotic tested.

### 2.4. Whole-Genome Sequencing

Genomic DNA was extracted from colonies growing on Coletsos media. Denaturation and DNA extraction from the MTBC strains were performed as previously described [16]. DNA quantity was assessed using the Qubit 2.0 fluorometer (Thermo Fisher Scientific, Waltham, MA, USA). WGS was performed using Illumina technology (MiSeq) (Illumina, San Diego, CA, USA). The Nextera XT DNA library preparation kit was used to prepare the library according to the manufacturer’s instructions. Fastq files (raw sequencing data) were submitted to the European nucleotide archive (see Table S1 for accession numbers). 

### 2.5. Variant Calling

We first discarded bases with a low-quality score (<30) and removed the adapters with Trim Galore, a wrapper of the Cutadapt program [17]. We mapped the obtained reads to the *M. tuberculosis* H37Rv reference genome (GenBank ID: NC_000962.3) with BWA-MEM [18,19]. We called SNPs (single-nucleotide polymorphisms) with BCFtools v 1.1 [20] with default parameters for bcftoolsmpileup and bcftools call. We filtered variants (bcftools filter) using the following threshold: minimum mapping quality of 20; minimum base quality at a position of 30; minimum read depth at a position of 20X; minimum percentage of reads supporting the call of 90%; and a minimum of 12 bases between two variants. We excluded the SNPs in previously defined repetitive regions (PPE and PE-PGRS genes) [21]. We considered the SNPs obtained as valid and used them for concatenated SNP alignments. We generated a consensus sequence with bcftools consensus using default parameters.

### 2.6. Typing and Genotypic Drug Resistance Prediction

We assigned the MTBC samples to the main phylogenetic lineages and sub-lineages and detected the mutations linked to drug resistance (first- and second-line) with TBProfiler 2.8.12 [4] using default parameters from the Fastq files.

### 2.7. Phylogenetic Analyses and Result Visualization

The alignment of 41,959 SNPs was used to construct a maximum likelihood phylogenetic tree with RAxML, using the GTRGAMMA model with 1000 bootstrap replicates and *M. canettii* (SRR10522763) as the outgroup. To evaluate the strain diversity of our collection, we plotted the world distribution of our MTBC strains (packages ”ggplot2” [22] and “scatterpie” in R [23]).

### 2.8. Accession Number(s)

The whole-genome sequences of the strains have been deposited in GenBank under BioProject PRJEB52390 (GenBank accession numbers ERS11871655 to ERS11871881).

### 2.9. Statistical Analysis

Student’s *t*-test and Fisher’s exact tests were performed when appropriate for comparisons. A *p*-value of <0.05 was considered significant.

## 3. Results

### 3.1. Characteristics of the Strains

Two hundred and twenty-seven MTBC strains isolated from pulmonary and extrapulmonary specimens during routine care at Avicenne Hospital between 2016 and 2021 were included in this study. Patients came from Africa (44%), Asia (27%), Europe (26%) and America (3%). The mean age of the patient was 36 years old, and men represented 70% of the population, which agreed with French TB epidemiology [24].

Strains isolated from extrapulmonary samples were associated with patients born in Asia (*p* = 0.001), whereas strains isolated from pulmonary samples were associated with patients born in Europe (*p* = 0.003). We did not find any association between sex and tuberculosis form (pulmonary or extrapulmonary) (*p* = 1).

### 3.2. Lineages Identification and Phylogenetic Analysis

**Figure 1:**
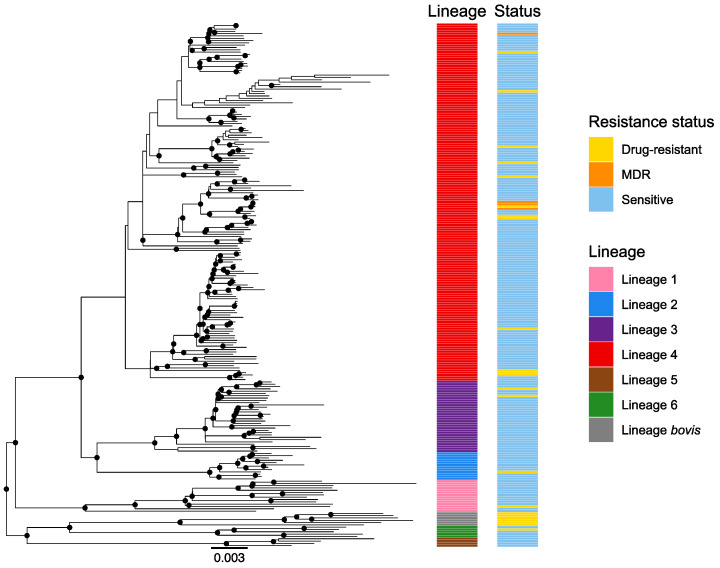
Global phylogeny of 227 whole-genome sequenced MTBC strains. The color codes are: pink—lineage 1, blue—lineage 2, purple—lineage 3, red—lineage 4, brown—lineage 5, green—lineage 6, gray—*M. bovis* lineage. The resistance status is represented for each strain with the following color code: blue—susceptible strains, yellow—strains resistant to one of the first-line drugs, orange—MDR strains (resistance to rifampicin and isoniazid). Only resistances to first-line TB drugs were considered here. MDR: multidrug-resistant.

All the described lineages were found in our collection with the exception of lineage seven (the Ethiopian lineage) (Figure 1). Euro-American lineage 4 was the most prevalent (68%), followed by the East-African-Indian lineage 3 (14%), the Indo-Oceanic lineage 1 (6%), the East Asian lineage 2 (5%), the West African lineages 5 (2%) and 6 (2%) and *bovis* lineage (3%) (Figure 1). To assess the lineage SNP diversity, we computed the minimum, maximum and mean number of SNPs among strains within each lineage. We found a higher SNP diversity within lineages 1, 6 and *bovis* lineage compared to the other lineages (Table S2).

Then, we compared the lineage distribution of our dataset with the lineage distribution of 214 samples studied by spoligotyping from the Bichat Hospital in Paris. We compared our results with only the samples of theirs that were studied during the same period (between 2015 and 2016) [24]. The lineage distributions were different (*p*<0.001). Compared to strains sampled in Paris (Bichat Hospital), our dataset comprised more lineage 3 strains (14% compared to 3%) and less lineage 2 (5% compared to 11%) and lineage 6 strains (2% compared to 5%) (pairwise comparisons using Fisher’s exact test).

Regarding antibiotic resistance, we did not detect any association between a specific lineage (lineage 1–6) and the resistance status of the strains (*p* = 0.976) (Figure 1). Additionally, we did not detect any association between sex and a specific lineage (*p* = 0.20).

**Figure 2:**
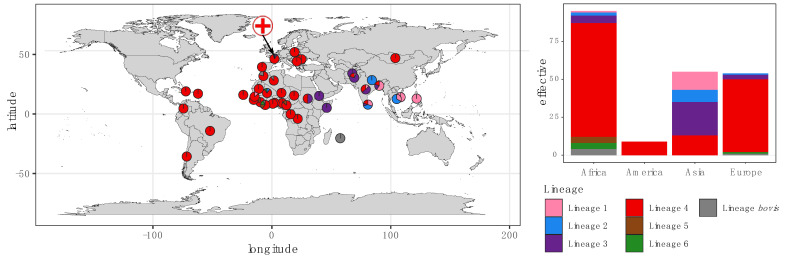
Global distribution of *M. tuberculosis* lineages by country of origin amongst the patients included in this study. Each color represents a distinct TB lineage. The symbol of a cross on the map represents the location of the Avicenne Hospital.

Our collection, sampled from a single hospital in France, was characterized by high patient diversity, with patients originating from 39 different countries (Figure 2 and Table S1). With the exception of Asia (Fisher’s exact test, *p* < 0.001), the lineage distribution for each continent is in agreement with the known global lineage distribution [3]. In our collection, the Euro-American lineage 4 was predominant in patients born in Africa, America and Europe, whereas patients from Asia also carried a large proportion of strains belonging to the lineages 1, 2 and 3 (Figure 2). Strains belonging to lineage 4 were significantly associated with patients born in Europe (*p* = 0.002) or Africa (*p* = 0.03), whereas strains belonging to lineages 1, 2 and 3 were significantly associated with patients born in Asia (*p* < 0.01). No association was found between resistance and country of origin (*p* = 0.98).

**Figure 3:**
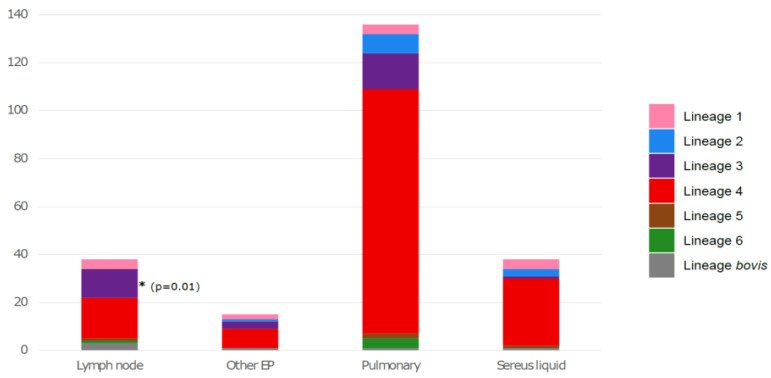
Distribution of *M. tuberculosis* lineages according to the origin of the sample. EP: extrapulmonary. The asterisk correspond to the significative association between the East-African-Indian lineage and lymph node TB.

Then, we considered the distribution of different lineages according to the type of sample from which the strains were taken in order to see if there was an association between a specific lineage and the clinical manifestations of TB. In this context, samples have been classified as follows: pulmonary (bronchial aspiration, bronchoalveolar fluid, sputum, gastric tube), lymph node, sereus fluids (cerebrospinal fluid, pleural fluid, peritoneal fluid, ascites fluid and pleural) and other extrapulmonary samples (bone, cutaneous, urine, genital and blood samples). Interestingly, the East-African-Indian lineage 3 was significantly associated with lymph node TB (*p* = 0.01) (Figure 3).

### 3.3. Drug-Susceptibility Testing

According to standard phenotypic DST, 87% (197/227) of isolates were susceptible to the four first-line drugs tested. Thirteen percent of the strains (30/227) were resistant to at least one first-line drug. Among them, only 1 was phenotypically RIF-resistant, 14 were only phenotypically INH-resistant and 11 were only phenotypically PZA-resistant, including 6 identified as *M. bovis*. Four strains (2%) were MDR-TB (resistant to both RIF and INH). The antibiotic phenotypic resistance rates of all the clinical strains were as follows: RIF, 2.2%; INH, 7.9%; EMB, 0.4%; PZA, 5.3%.

### 3.4. Comparison of WGS and Phenotypic DST Results

Interpretable results of WGS (depth coverage ≥30X) were available for all MTBC isolates. The accuracy of results from Illumina sequencing was in line with phenotypic DST results, as the global concordance was 97% (for 221/227 strains, we obtained concordant results between the two methods for the four first-line anti-TB drugs). The sensitivity of WGS for predicting RIF and INH resistance was 100%, and the specificity of WGS for predicting RIF and INH susceptibility was 100% (Table 1). For EMB, the sensitivity and the specificity of WGS were 100% and 99.1%, respectively. Two phenotypically EMB-susceptible isolates were found genetically resistant because of a mutation in the *embB* gene (A313V and G406A variants) (Table 1). Regarding PZA, the sensitivity of WGS for predicting resistance was only 66.7%, while the specificity was 100% (Table 1). Indeed, phenotypic DST and WGS were discordant for four phenotypically resistant strains, while no nonsynonymous mutation was detected in the *pncA* and *panD* genes by WGS (only one synonymous mutation was found in the *pncA* gene for one strain).

WGS not only detects resistance to first-line anti-TB drugs but also second-line anti-TB drugs. The results of WGS show that 15% of the strains (35/227) were resistant to at least one of the second-line anti-TB drugs. More precisely, mutations in the *gyrA* gene conferring resistance to fluoroquinolones were detected for six strains. A mutation in the *tlyA* gene (N236K) conferring resistance to capreomycin was found in one strain. Mutations in the *ethA*, *ethR* or *fabG1* genes conferring resistance to ethionamide were found for 14 strains. Mutations in the *gid, rrs* and *rpsL* genes conferring resistance to streptomycin were found for 16 strains.

## 4. Discussion

Poverty rate and TB incidence are correlated at all scales (intra-regional, regional, national) [25]. The Seine-Saint-Denis, a French department located in the urban area of Paris, appears to be much more affected than other French metropolitan departments. In this department, a particularly high proportion of foreigners and inhabitants reporting difficult living conditions has been associated with a higher incidence of TB [14]. The laboratory of Avicenne Hospital, located in Seine-Saint-Denis, isolates a hundred MTBC strains each year for which drug susceptibility and resistance are phenotypically assessed. To evaluate the use of WGS in hospital laboratories, we sequenced 227 randomly selected strains. We found a high level of phylogenetic diversity, mirroring the high diversity of origins of the population in the department. Despite a few discrepancies that need to be further explored in the future, the WGS assessment of resistance largely agreed with the phenotypic DST (97%).

### 4.1. Local Epidemiology and Lineage Diversity

Our study focused on a single urban laboratory in a department with an incidence rate three times higher than in other French departments. We found a high level of diversity for the strains isolated in our laboratory, with, as expected, a majority of strains (68%) belonging to the Euro-American lineage. Interestingly, when mapped according to patients’ geographical origins, the lineage distribution is in line with the known global TB diversity (Figure 2) [6,26,27]. These results suggest that the patients were infected in their country of origin by local MTBC strains. Indeed, because TB can be a latent disease for decades, the population in cosmopolitan centers could have been contaminated by MTBC strains in their region of birth. As a consequence, the host’s region of origin is predictive of the strain of TB carried by the host, and this association between host and pathogen populations appears to be highly stable [7]. The local geographic diversity of MTBC lineages was probably dictated by the composition of the local immigrant population. However, the transmission of a circulating strain belonging to a specific lineage within a community cannot be excluded. Our lineage distribution was different from that found in the study of Pierre-Audigier et al. [24]. This discrepancy could be explained by the ethnical differences of the populations living in these areas. A study on a larger scale with strains from different regions of France could help us to better understand the influence of ethnicity on the proportion of lineages found in each territory.

Determining *M. tuberculosis* lineage is essential for designing TB control due to the impact of specific genotypes on disease outcome [9]. Many studies have shown that phylogenetic lineages of MTBC can be associated with different clinical manifestations [28,29,30,31]. In our study, we found that the Euro-American lineage (lineage 4) was significantly less likely to cause extrapulmonary TB. We also found that the East-African-Indian lineage (lineage 3) was associated with lymph node TB, as previously described by Khandkar et al. [32]. This result could possibly indicate that the East-African-Indian strain has intrinsic virulence factors that must be investigated. It may also be a reflection of the higher prevalence of lymph node TB in the Asian population, where lineage 3 is the most prevalent genotype causing lymph node TB in this region [33].

### 4.2. Genomic Versus Phenotypic Approach for Diagnosing Drug Susceptibility and Resistance

The advantage of WGS is that it represents an “all-in-one” tool that helps us to investigate TB transmission dynamics to improve TB control, as previously reported [34,35,36], but it also addresses the question of drug susceptibility. In our study, the antibiotic phenotypic resistance percentage of 13% for all the clinical strains was concordant with the national resistance rate data concerning first-line TB drugs [37]. It is important to note that the low number of drug-resistant MTBC strains included in this study did not allow an accurate calculation of performances, but our estimates in terms of susceptibility and specificity give an idea of the performance of WGS. Analytical performances of WGS for the detection of resistance to the two major anti-TB drugs, RIF and INH, in comparison with DST, were excellent (100%). These performance estimates were consistent with previously published results [3,38,39]. However, despite a large agreement between DST and WGS in diagnosing resistance (97%), we found four phenotypically resistant but genetically susceptible strains and two phenotypically susceptible but genetically resistant strains. One possible cause of discrepant results observed between DST and WGS is the coexistence of drug-susceptible and drug-resistant isolates in the population [40,41]. This mechanism, referred to as heteroresistance, is common in MTBC [41,42,43]. 

Concerning PZA, the ability of WGS to predict phenotypic resistance was problematic in our study. We found four strains to be phenotypically PZA-resistant, whilst no mutation was detected in the *pncA* gene. False PZA resistance may explain these results, which is not surprising, since DST is technically challenging and not perfectly accurate for PZA resistance detection, with inadequate reproducibility [44,45,46]. There is a general view that the determination of phenotypic susceptibility to PZA is more complex for PZA than for other anti-TB drugs. Indeed, the growth of *M. tuberculosis* under acidic conditions (pH 5.5 to 6.0) is required for conventional DST of PZA, making this test challenging and problematic due to the poor growth of *M. tuberculosis* under these acidic conditions. Conventional DST of PZA is challenging and problematic due to the poor growth of *M. tuberculosis* under the acidic conditions (pH 5.5 to 6.0) required for optimal drug activity [45]. An increase in the pH in the test medium, caused by bacterial overinoculation, can give false resistance results [45,47]. It makes PZA a weak test for comparisons between phenotypic and genotypic results. Our results agree with studies suggesting that MGIT 960 has shown incidents of false-positive PZA resistance [44,48]. In a study evaluating the use of NGS on 88 MTBC strains to improve detection of PZA resistance, concordance between next-generation sequencing (NGS) and MGIT results was 93%, and NGS revealed several new and mixed-strain mutations [43]. Interestingly, for one of the PZA-resistant isolates, a new mutation in the gene *rpsA* (Gly17Ala) was found. The identification of nonsynonymous *rpsA* mutations in both PZA-susceptible and PZA-resistant isolates also implies that further studies are needed in order to determine the role of *rpsA* in PZA resistance [49]. For two phenotypically EMB-susceptible isolates, we found two mutations in the *embB* gene (A313V and G406A), a gene conferring resistance. The variant *embB* A313V has been found in two EMB-susceptible isolates [50] and in one resistant isolate [51]. The variant *embB* G406 elevates the MIC of EMB, but mostly at sub-threshold levels [52]. This low-level phenotypic resistance could explain the discrepancy observed for EMB. Indeed, some mutations confer “low-level” resistance in MTBC, resulting in ambiguous results from phenotypic tests and discrepancies between phenotypic and genetic analysis [3].

Contrary to standard phenotypic tests conducted on a daily basis in hospital laboratories, WGS offers the opportunity to determine resistance to second-line anti-TB drugs. In our study, we found that 15% of the strains were resistant to at least one of the second-line anti-TB drugs. We did not compare these results with DST, as the method was not implemented in the laboratory for the second-line anti-TB drugs. Determination of this resistance is interesting in the clinical management of MDR-TB strains, providing considerable information when compared with current routine methods [3]. WGS also offers the opportunity to reduce the delay in obtaining results. Phenotypic tests take several weeks to obtain results, which leads to a delay in the adaptation of TB treatments. In our study, we estimated a one-week delay in obtaining results from using WGS that is in agreement with other studies showing that WGS obtains TB drug resistance profiles an average of 9 days earlier than phenotypic tests for first-line anti-TB drugs [53,54].

Overall, WGS is an excellent tool, but the current discrepancies between genotype and phenotype for EMB and PZA need to be clarified, and at present, identifying true MTBC drug resistance still remains complex.

## 5. Conclusions

In this study using WGS, we established the circulating lineages and the drug-susceptibility trends in a hotspot region of TB. From a single sampling location, we found a high level of lineage diversity explained by the diversity of birth origins of patients living in this area. Due to its high resolution and its “all-in-one” solution, which is needed to guide clinical decisions, WGS in routine microbiology laboratories provides an essential tool to better understand local TB epidemiology and MTBC evolution.

## Figures and Tables

**Table 1:** Discrepancies between whole-genome sequencing (WGS) and phenotypic drug-susceptibility testing (DST).

Drug		Phenotypically Resistant			Phenotypically Susceptible				Consistency (%)		WGS			
		Genetically Resistant	Genetically Susceptible		Genetically Susceptible	Genetically Resistant	Undetected Variants				Sensitivity (%)	Specificity (%)	PPV (%)	NPV (%)
RIF		5	0		222	0	NA		100		100	100	100	100
INH		18	0		209	0	NA		100		100	100	100	100
EMB		1	0		224	2	*embB* A313V (one strain) *embB* G406A (one strain)		99.1		100	99.1	33.3	100
PZA		8	4		215	0	NA		98.2		66.7	100	100	98.2

RIF, rifampicin; INH, isoniazid; EMB, ethambutol; PZA, pyrazinamide; WGS, whole-genome sequencing; NPV, negative predictive value; PPV, positive predictive value. NA, not applicable.

## Data Availability

Not applicable.

## Supplementary Materials

The following supporting information can be downloaded at: https://www.mdpi.com/article/10.3390/microorganisms10081586/s1, Table S1: recapitulative table of the samples used in this study; Table S2: genetic diversity within lineages.
